# Comparison of end-to-side hand-sewn and side-to-side stapled cervical esophagogastric anastomosis in patients with lower thoracic esophageal cancer undergoing transhiatal esophagectomy: an Iranian retrospective cohort study

**DOI:** 10.1186/s12876-020-01393-x

**Published:** 2020-07-31

**Authors:** Seyed Ziaeddin Rasihashemi, Ali Ramouz, Samad Beheshtirouy, Hassan Amini

**Affiliations:** 1 Department of Cardiothoracic Surgery, Tabriz University of Medical Sciences, Tabriz, Iran; 2 Department of General, Visceral and Transplantation Surgery, Heidelberg, Germany; 3 Stem Cell Research Center, Tabriz University of Medical Sciences, Tabriz, Iran; 4 Department of General Surgery, Imam Reza Hospital, Tabriz University of Medical Sciences, Tabriz, Iran

**Keywords:** Esophageal cancer, Side-to-side stapled cervical esophagogastric anastomosis, End-to-side hand-sewn anastomosis, Anastomotic leakage, Anastomotic stricture

## Abstract

**Background:**

Controversies in terms of efficacy and postoperative advantages surround stapled esophagogastric anastomosis compared with the hand-sewn technique as a treatment for patients with esophageal cancer. The purpose of this study was to compare the clinical outcomes of hand-sewn end-to-side esophago-gastrostomy and side-to-side stapled cervical esophagogastric anastomosis after esophagectomy for the aforementioned patients.

**Methods:**

This retrospective cohort study involved examining the medical records of 433 patients who underwent transhiatal esophagectomy for esophageal cancer from March 2010 to March 2016. All the patients were operated using end-to-side hand-sewn esophago-gastrostomy and side-to-side stapled cervical esophagogastric anastomosis. 409 of the patients received a year’s worth of follow-up evaluations. All the cases were revisited in 2 weeks as well as in four, eight, and 12 months after surgery. The patients were assessed in terms of postoperative outcomes, including reflux symptoms, anastomotic leakage and stricture, and the need for anastomotic dilatation.

**Results:**

Hand-sewn anastomosis was carried out in 271 (62.5%) patients, whereas stapled anastomosis was performed in 162 (37.4%) patients. The mean operative times were 214.46 ± 84.33 min and 250.55 ± 43.31 min for the stapled and hand-sewn anastomosis groups, respectively (*P* = 0.028). The two groups showed no significant differences with respect to stays in intensive care units and hospitals. Postoperatively, 38 (14.67%) cases of anastomotic leakage were detected in the hand-sewn anastomosis group, with incidence being significantly higher than that in the stapled anastomosis group (8 cases or 5.33%; *P* = 0.002). Anastomotic stricture occurred less frequently in the patients who underwent stapled anastomosis (*P* = 0.004). Within the one-year follow-up period, the patients treated via hand-sewn anastomosis more frequently required anastomotic dilatation (*P* = 0.02).

**Conclusion:**

Side-to-side stapled cervical esophagogastric anastomosis may reduce operation times and decrease the rates of anastomotic leakage, anastomotic stricture, and anastomotic dilatation in patients with lower thoracic esophageal cancer undergoing transhiatal esophagectomy.

## Background

Esophageal cancer is one of the most prevalent and multifaceted gastrointestinal malignancies and the sixth leading cause of mortality among cancers [1, 2]. Various methods have been introduced as a mainstay of treatment, including surgical procedures and non-surgical palliative approaches, but the current standard for the management of esophageal cancer is esophagectomy [3–5]. Esophagogastric anastomosis, including the popular variants hand-sewn and stapled anastomoses, is the most critical procedure during esophagectomy [6, 7]. Regardless of surgical approach, nonetheless, preventing anastomotic complications is necessary to minimize early morbidity and improve intervention outcomes [6].

Postoperative complications subsequent to esophagogastric anastomosis may lead to life-threatening situations, including anastomotic leakage, anastomotic stricture, and other rare complications, such as fistulas and abscesses. Anastomotic leakage occurs in more than 10% of patients undergoing esophagogastric anastomosis, with the condition accompanied by some complications, such as mediastinitis, nourishing discomfort, and anastomotic stricture as well as less common complications, including cervical osteomyelitis [6, 8, 9]. An important requirement therefore is to choose a surgical procedure that can accurately and effectively prevent and reduce post-anastomotic complications. An automatic stapling for intestinal anastomosis was introduced by Berthold et al. in 1980. Stapled techniques have been presented in the past years as a means of minimizing the risk of anastomotic leakage and stricture [10–12]. Collard et al. used linear stapling devices in 1998 to carry out esophagogastric anastomosis [13], and Orringer applied structural modifications to previously developed techniques to improve results [14], but whether the improved versions are superior remains a matter of debate.

This deficiency is exacerbated by the fact that although some studies, including meta-analyses and systematic reviews, have been conducted to compare the effectiveness of hand-sewn and stapled anastomosis techniques (through the use of linear or circular staplers), the superiority of one approach over the other remains a controversial issue [15, 16]. To address this matter, we compared the operation times and postoperative outcomes of transhiatal esophagectomy and cervical esophagogastric anastomosis performed via hand-sewn and stapled techniques over a year of follow-up. The specific issues compared were symptoms, anastomotic leakage, anastomotic stricture, and the need for anastomotic dilatation in patients.

## Methods

An initial sample of 575 consecutive patients with resectable esophageal cancer undergoing esophagectomy and regional lymphadenectomy from March 2010 to March 2016 were screened for possible inclusion in the study. These patients were those admitted into the Department of Thoracic Surgery at Imam Reza Hospital in Tabriz, Iran (the referral hospital in the northwestern region of the country).

The exclusion criteria were as follows: (i) patients who underwent esophagogastric anastomosis via circular stapling (*n* = 68), (ii) patients who had reconstruction using colonic or jejunal interposition (*n* = 44), (iii) patients with incomplete medical records (*n* = 10), and (iv) patients subjected to Ivor Lewis, McKeown, or thoracoabdominal esophagectomy (*n* = 20). On these bases, 142 patients were excluded, and the remaining 433 were enrolled in this study. The study protocol was approved by the Regional Ethics Committee headed by the Vice-Chancellor of Research and Development at Tabriz University of Medical Sciences (Ethical approval number: 91/1–1/4). Written informed consent was obtained from each patient during questionnaire administration for the collection and analysis of applicable clinical data. The study protocol conforms to the ethical guidelines of the 1975 Declaration of Helsinki, as reflected in a priori approval by the institution’s human research committee.

Demographic information, including age, sex, preoperative clinical signs and symptoms, and intraoperative surgery-related variables, such as operation time, anastomotic approach, pathological stage, and postoperative outcomes, were extracted from medical records. The patients were evaluated postoperatively for anastomotic leakage and stricture on the 12th month of follow-up.

### Surgical procedures

**Fig. 1:**
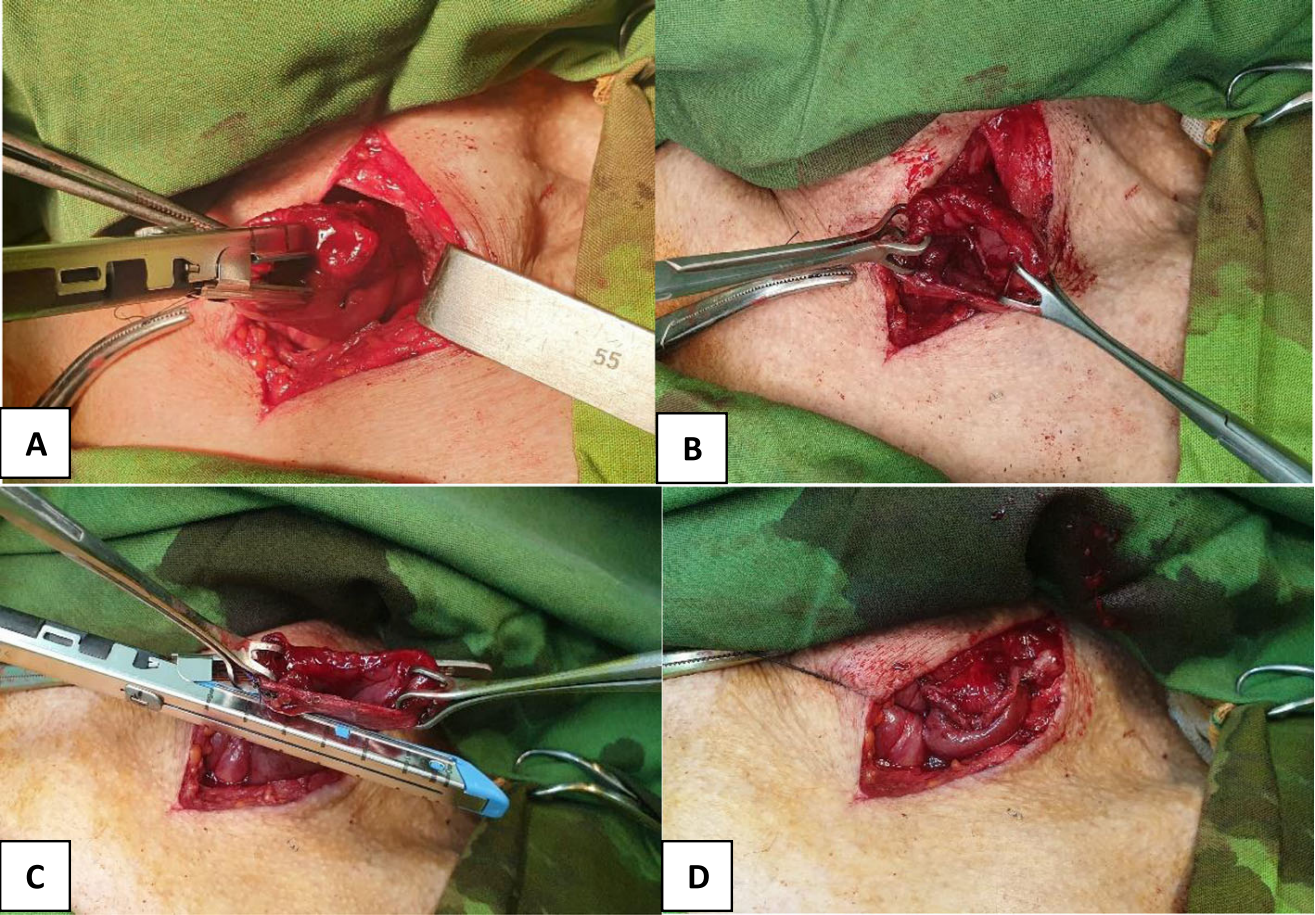
**a**: The posterior wall of esophagus and the posterior wall of gastric conduit aligned and two stay sutures applied. **b**: The posterior wall of the anastomosis constructed by using an Endo GIA™ loaded with 60 blue cartridge fired vertically. **c** and **d**: The lateral sides of anastomosis pulled up by two backups and a 60-mm linear stapler (Endo-GIA60–3; Covidien) fired horizontally to close the anterior wall of the anastomosis

The patients were subjected to esophagectomy and regional lymphadenectomy through a transhiatal blunt dissection performed with the patients in the supine position. The cervical esophagus was then mobilized through an oblique incision on the left side of the neck, parallel to the anterior border of the sternocleidomastoid muscle. A 4 cm wide gastric tube was pulled through the posterior mediastinum for cervical esophagogastric anastomosis. To support short-term enteral nutrition, a jejunostomy tube was inserted for all the patients during laparotomy. The cervical esophagogastric anastomosis was performed via the hand-sewn or stapling technique. In the manual (hand-sewn) method, the end of the cervical esophagus was sutured to the anterior wall of the pulled-up stomach in the neck via interrupted two-layer suturing with 3–0 Vicryl for the inner layer and 3–0 Prolene for the outer layer. In the stapled anastomosis, an Endo GIA™ loaded with 60 blue cartridges (Covidien-Medtronic, Minneapolis, MN, USA) were used to perform anastomosis of the posterior wall of the esophagus to the posterior wall of the gastric conduit. The anterior wall was then constructed using another 60 mm linear stapler (Endo GIA60; Covidien) (Fig. 1).

All the patients were transferred directly to the intensive care unit (ICU) with a nasogastric tube and a Penrose drain attached to the neck. Enteral feeding via the jejunostomy tube was initiated on the third day after surgery. After operation, the patients were evaluated for anastomotic leakage in a radiographic contrast study on days 6 to 9. The anastomotic leakage was diagnosed on the basis of saliva leakage through the cervical drains or the presence of extraluminal contrast on the esophagogram or computed tomography (CT) scans of the neck. Patients whose esophagogram manifested leaks with little to no clinical presentation were classified as having minor leakage, whereas the presence of leakage symptoms, regardless of contained or uncontained appearance on the radiographs (e.g., as is the case with conduit necrosis requiring reoperation), was considered reflective of major leakage. In the absence of leakage, oral feeding was started with water and a semiliquid diet. However, in the presence of even subtle signs of cervical wound infection and considerable saliva secretion from the embedded Penrose drain or anastomotic leakage detected via contrast study, oral feeding was postponed, and the cervical wound was reopened to about 2 to 3 cm wide to establish drainage.

### Follow-up

Patients were followed up regularly, with history and physical exams conducted 2 weeks after surgery, then every 4 months for the first year. If difficulty in swallowing developed, endoscopic evaluation was performed. A diagnosis of benign anastomotic stricture was made when a 10 mm flexible endoscope could not be passed through the anastomosis. Histological examination was conducted to rule out the occurrence of malignant stricture. Anastomotic dilatation was performed under general anesthesia to treat symptomatic patients.

### Statistical analyses

Continuous variables were expressed as means±SD. For the quantitative data, normality was evaluated using the Kolmogorov–Smirnov test, and Mauchly’s W test was carried out to identify the covariance matrix. A repeated-measures with control covariates test was run in the Statistical Package for the Social Sciences (SPSS version 17). A chi-square test was performed to compare two qualitative variables. The McNemar test was used to compare each operational duration with the base time, and Cochran’s Q test was implemented to compare the dependent variables in terms of varying times of operation. A *P*-value of < 0.05 was considered indicative of statistical significance. All the results were expressed as frequencies (percentages) for the qualitative variables and means±SD for the quantitative variables.

## Results

### Perioperative outcomes

Among the 433 consecutive patients with esophageal cancer, 271 (62.5%) belonged to the hand-sewn anastomosis group, and 162 (37.4%) were assigned to the stapled anastomosis group. Of the subjects, 248 (57.3%) were male, and 185 (42.7%) were female. The demographic data and clinical characteristics of the patients are presented in Table 1.

**Table 1:** Patients’ demographic data and preoperative symptoms

Anastomosis type		Hand-sewn (*n* = 271)	Stapled anastomosis (*n* = 162)	*P* value
Sex	Female	85 (31.36%)	100 (61.72%)	0.08
	Male	186 (68.64%)	62 (38.28%)	
Age (year)		65.44 ± 19.11	62.62 ± 20.19	0.338
Preoperative complaints	Dysphagia	271 (100%)	162 (100%)	1
	Heart burn	130 (47.97%)	87 (53.7%)	0.24
	Weight loss	100 (36.9%)	54 (33.33%)	0.45
	Odynophagia	75 (27.67%)	68 (41.97%)	0.482
Tumor type	SCC	250 (92.25%)	135 (83.33%)	0.004
	Adenocarcinoma	21 (7.74%)	27 (16.66%)	
Stage of tumor	1	54 (19.92%)	19 (11.72%)	0.01
	2	140 (51.66%)	78 (48.14%)	
	3	77 (28.41%)	65 (40.12%)	

Patients’ demographic data and preoperative symptoms patients underwent hand-sewn and stapled anastomosis groups

The groups showed no significant differences in terms of age, gender, and preoperative clinical signs and symptoms. Despite the high incidence of squamous cell carcinoma in both groups, the rate of esophageal adenocarcinoma was significantly higher in the stapled anastomosis group (*P* = 0.004). Although the groups significantly differed in tumor stage at the time of operation (*P* = 0.01), the post hoc analysis revealed no difference in this respect between the hand-sewn and stapled anastomosis patients.

Table 2 shows the durations of operation, ICU and hospital stays, and postoperative morbidity and mortality rates of the patients. Compared with the hand-sewn anastomosis group, the stapled anastomosis group had significantly shorter operation times (*P* = 0.028). Nevertheless, no significant differences in ICU and hospital stays and perioperative complications other than anastomotic leakage were found between the groups. All the patients were evaluated for postoperative anastomotic leakage by water-soluble esophagogram or neck CT with oral contrast. The incidence of anastomotic leakage in the hand-sewn and stapled anastomosis groups were seen in 38 (14.02%) and eight (4.93%) cases, respectively. The incidence was significantly lower in the stapled group (*P* = 0.002). Overall, 44 (10.1%) patients had minor leaks and received conservative management, which consisted of delays in oral intake (usually 1 week), cervical wound drainage, enteral nutrition via a jejunostomy tube, and selective antibiotic administration. Among the nine patients who underwent reoperation, four (1.5%) were in the hand-sewn anastomosis group, and five (3%) belonged to the stapled anastomosis group. Two (0.41%) patients with considerable leakage and subsequent conduit necrosis had revisional surgery, which included a resection of the gastric conduit and construction of cervical esophagostomy.

**Table 2:** Patients’ postoperative findings

Anastomosis type		Hand-sewn (*n* = 271)	Stapled anastomosis (*n* = 162)	*P* value
Operation time (minute)		250.55 ± 43.31	214.46 ± 84.33	0.028
Morbidity	Anastomotic leakage	38 (14.02%)	8 (4.93%)	0.002
	Minor	37 (13.6%)	7 (4.32%)	
	Major	1 (0.32%)	1 (0.61%)	
	Pneumonia	20 (7.38%)	15 (9.25%)	0.1
	Pleural effusion	21 (7.74%)	11 (6.79%)	0.63
	Pneumothorax	13 (4.79%)	7 (4.32%)	0.81
	Chylothorax	5 (1.84%)	4 (2.46%)	0.23
	ARDS	3 (1.1%)	0	0.24
	RLN injury	4 (1.47%)	1 (0.61%)	0.38
	Arrhythmia	9 (3.32%)	11 (6.79%)	0.07
	Wound infection	5 (1.84%)	3 (1.85%)	0.63
	Others	13 (4.79%)	12 (7.4%)	0.054
Mortality	Hospital mortality	7 (2.58%)	4 (2.46%)	0.60
	30-day mortality	1 (0.36%)	1 (0.61%)	0.57
	90-day mortality	2 (0.73%)	0	0.48
Hospital stay (Days)		19.5 + 7.2	18.5 + 7.7	0.17
ICU stay (Days)		9.7 + 7.5	9.1 + 7.2	0.42
Malignant anastomotic Stricture		3 (1.1%)	2 (1.23%)	0.61
Pathologic positive margin		2 (0.73%)	2 (1.23%)	0.48
Reoperation	Chylothorax	1 (0.36%)	2 (1.23%)	0.31
	Tracheal injury	2 (0.73%)	0	0.39
	Conduit necrosis	1 (0.36%)	1 (0.61%)	0.60
	Non anastomotic leak	0	2 (1.23%)	0.13

Patients’ postoperative outcomes in hand-sewn and stapled anastomosis groups

Hospital mortality rates were 2.58% (*n* = 7) and 2.46% (*n* = 4) in the hand-sewn and stapled anastomosis groups, respectively (*P* = 0.60). Of 2 patients who died in 30-day after hospital discharge, one (0.56%) received hand-sewn esophago-gastrostomy and the other (0.061%) underwent stapled cervical esophagogastric anastomosis (*P* = 0.57). Two (0.73%) patients belonging to the hand-sewn esophago-gastrostomy group died within 90 days after the operation (*P* = 0.48). One death due to anastomotic leakage occurred in each group.

### Postoperative outcomes

Among the 433 cases, 24 were excluded from the 12-month follow-up evaluation period because of postoperative mortality, anastomotic recurrence, and positive esophageal proximal resection margin (Table 2). This left 409 patients for assessment as regards postoperative complications, including reflux symptoms, benign anastomotic stricture, and the need for anastomotic dilatation at the 12th month of follow-up. All the patients underwent serial clinical examinations and appropriate workup during the second week, as well as in the fourth, eighth, and 12th months after operation.

**Fig. 2:**
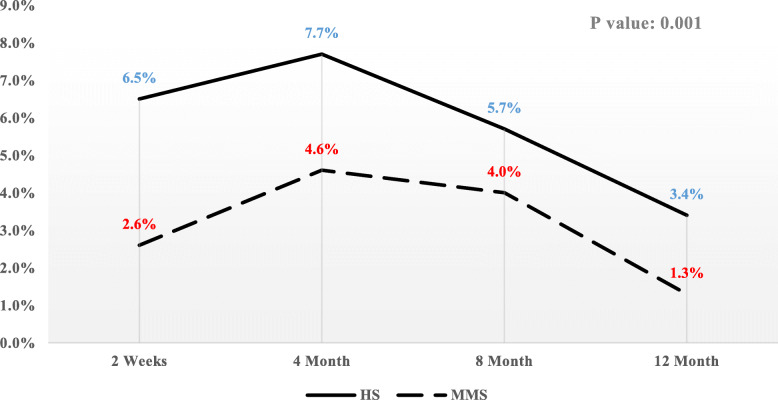
Prevalence pattern of reflux symptoms in hand-sewn and stapled anastomosis groups in over the 12-month follow-up period

The prevalence of reflux symptoms (heartburn and regurgitation) during the follow-up is depicted in Fig. 2. These symptoms occurred less frequently in patients with stapled anastomosis (*P* = 0.001), but pattern decreased in both groups at the final steps of the follow-up period. Changes in reflux prevalence were higher in the hand-sewn anastomosis group during the early stages of the follow-up (*P* = 0.004), but prevalence was significantly eliminated within the one-year follow-up (*P* = 0.02).

Upper gastrointestinal barium swallow and subsequent upper gastrointestinal endoscopy were performed to investigate anastomotic stricture in the patients with complaints of dysphagia, odynophagia, and retrosternal pain. Data on the prevalence of anastomotic stricture during the follow-up period are summarized in Table 3. During the follow-up, the groups were compared in terms of different time points via Cochran’s Q test, which revealed that the prevalence of anastomotic stricture was significantly higher in the hand-sewn anastomosis group than in their stapled anastomosis counterparts (*P* = 0.004 vs 0.263). The mixed model test demonstrated that changes in such prevalence were significantly fewer in the stapled group than the hand-sewn group (*P* = 0.029).

**Table 3:** Prevalence pattern of anastomotic stricture

Anastomosis stricture		Status	Two weeks	4 Month	8 Month	12 Month	*P* value ^*^
Anastomosis technique	Hand-sewn	Present	0 (0%)	28 (10.8%)	37 (14.2%)	23 (8.8%)	0.004
		Not present	0 (0%)	231 (89.2%)	222 (85.8%)	236 (91.2%)	
		McNemar	Base	0.008	0.083	0.083	
	Stapled anastomosis	Present	0 (0%)	9 (6%)	8 (5.3%)	4 (2.6%)	0.263
		Not present	0 (0%)	141 (94%)	142 (94.7%)	146 (97.4%)	
		McNemar	Base	0.005	0.083	0.046	
Statistical Tests	Chi-Square		1	0.07	0.003	0.01	
	Mixed Model		Pv Group = 0.029, Pv Time = 0.235				

*Cochran’s Q Test

Prevalence pattern of anastomotic stricture in patients underwent hand-sewn and stapled anastomosis groups

The prevalence pattern of anastomotic dilatation is illustrated in Table 4. All the patients with symptomatic anastomotic stricture underwent esophageal dilatation guided by Savary–Gilliard bougie dilators during rigid esophagostomy performed under general anesthesia. The patients who were subjected to hand-sewn anastomosis required significantly more dilatation than did the stapled anastomosis group at different time points during the follow-up period (*P* = 0.048 vs 0.273). Compared with the hand-sewn anastomosis group, the stapled anastomosis group required fewer changes for intervention, as determined in the mixed model test conducted during the follow-up period (*P* = 0.021).

**Table 4:** Prevalence pattern of intervention for anastomotic dilatation

Need for dilatation		Status	4 Month	8 Month	12 Month	*P* value ^*^
Anastomosis technique	Hand-sewn	Present	12 (4.6%)	13 (5%)	16 (6.1%)	0.048
		Not present	247 (95.4%)	246 (95%)	243 (93.9%)	
		McNemar	Base	0.893	0.317	
	Stapled anastomosis	Present	4 (2.6%)	11 (7.3%)	3 (2%)	0.273
		Not present	146 (97.4%)	139 (92.7%)	147 (98%)	
		McNemar	Base	0.564	0.157	
Statistical Tests	Chi-Square		0.239	0.227	0.04	
	Mixed Model		Pv Group = 0.021, Pv Time = 0.473			

*Cochran’s Q Test

Prevalence pattern of intervention for anastomotic dilatation in patients underwent hand-sewn and stapled anastomosis groups

## Discussion

The stomach is a good alternative for patients with esophageal cancer undergoing esophagectomy. Traditionally, the accepted standard treatment for operable esophageal carcinoma is resection of the esophagus and lymph nodes with gastric pull-up and construction with cervical or intrathoracic esophagogastric anastomosis [6, 10, 17]. The main complications occurring after esophagectomy and esophagogastric anastomosis are anastomotic leakage and stricture, which may affect patients’ quality of life [17]. Recent studies have shown that anastomotic leakage and the type of mechanical stapler used in surgical procedures are critical to the development of anastomotic stenosis [18, 19].

The present research was conducted on 409 patients undergoing esophagectomy via end-to-side hand-sewn and side-to-side stapled cervical esophagogastric anastomoses. The results showed a significantly high prevalence of cervical esophagogastric anastomotic leakage following transhiatal esophagectomy through manual anastomosis. In a retrospective study Cooke et al. indicated that 1133 patients undergoing esophagectomy followed by esophagogastric anastomosis showed a significant reduction in postoperative complications and the prevalence of problems in anastomotic construction using mechanical anastomosis [7]. The literature reflected that the incidence rate of cervical esophagogastric anastomotic leak falls between 9 and 14% but that rates of 15 to 25% are commonly reported with respect to hand-sewn anastomosis [5, 20]. In the present study, the prevalence of anastomotic leakage in patients undergoing stapled and hand-sewn anastomoses were 4.9 and 14.02% respectively. These findings may be more acceptable than the levels derived in other studies. It is understandable that the difference in leakage rates between anastomotic techniques is difficult to distinguish given that leakage incidence is less than 3% when anastomosis is performed by experienced thoracic surgeons [5]. Similar to our results, those of Mishra et al. showed that the rate of anastomotic leakage was significantly higher in patients undergoing hand-sewn anastomosis than in patients treated via linear stapled anastomosis [21].

Laterza et al. compared manual and mechanical anastomoses and found that patients treated using the latter exhibited a high prevalence of anastomotic leakage and benign stricture [22]. Other randomized controlled trials revealed a higher prevalence of anastomotic leakage and anastomotic stricture in manually operated individuals, suggesting the superiority of mechanical anastomosis as a technique for esophagogastric anastomotic construction [23–25]. Even in intrathoracic esophagogastric anastomosis where linear stapling is conducted, a significant decrease in anastomotic leakage and stricture was observed compared with the levels occurring under hand-sewn anastomosis [19].

Sugimura et al. used a modified Collard technique and a linear stapler to construct the posterior wall in anastomosis and closed the anterior wall using the linear stapler twice. The authors showed that anastomotic leakage was less frequent in the modified Collard group than in the hand-sewn group but that the difference was not significant. Anastomotic stenosis occurred to a significantly lower extent in the modified Collard group, and the period between esophagectomy and initial dilatation was significantly shorter in the hand-sewn anastomosis group [26]. Similarly, Ishibashi et al. performed triple-stapled quadrilateral anastomosis to create esophagogastric anastomosis and reported no significant anastomotic leakage and stricture [27].

Some reviews indicated no significant difference between hand-sewn and stapled anastomosis techniques in terms of the prevalence of anastomotic stricture. However, our results showed a decreasing pattern in the rate of anastomotic stricture during the follow-up period in the stapled anastomosis group compared with the rate observed in the manual anastomosis patients. Comparably, Cooke et al. discovered a significant reduction in the prevalence of postoperative complications and morbidity in patients for whom mechanical anastomosis was carried out [7]. Price et al. found that although an anastomotic site was irrelevant to the likelihood of postoperative complications, such as anastomotic leakage and stenosis, patients treated via manual anastomosis experienced a higher incidence of anastomotic leakage and stricture [25].

The current study uncovered that the prevalence of benign anastomotic stricture during the 12th month of follow-up was significantly higher in the hand-sewn anastomosis group than in their stapled counterparts. This led us to conclude that mechanical anastomosis plays an important role in reducing the incidence of postoperative complications by creating a wider anastomotic space than that achieved with a hand-sewn technique.

There are several studies showing an increase in reflux symptoms following esophagectomy and gastric pull up in patients with esophageal cancer [28–30]. The current study uncovered that the prevalence of reflux symptoms during the 12th month of follow-up was significantly higher in the hand-sewn anastomosis group than in their stapled counterparts. However, Ercan et al. reported no significant difference between stapled and hand sewn anastomoses in reflux symptoms. Despite the increase in the diameter of the anastomosis, the probability of reflux did not increase in the patients of their study [31]. Sugimura and colleagues showed that frequency of reflux esophagitis tended to be lower in the mechanical group than in the hand-sewn group prior to propensity score matching [26]. In our study, the length of the remaining cervical esophagus was longer than the hand-sewn group in order to create a proper anastomosis in the stapled group. Therefore, the location of the anastomosis was at the entrance to the chest; while in the hand-sewn method, the anastomotic location was performed approximately 3 cm below the cricopharynx level. Consequently, we hypothesized that the length of esophagus remnant may be a major factor contributing to the reduced prevalence of reflux symptoms in patients with stapled anastomosis.

We also evaluated endoscopic bougie dilatation to relieve benign anastomotic stenosis and recurrent dysphagia within the 12-month follow-up. The statistical results showed a significant difference between the patients who required esophageal dilatation. In a similar vein, Sugimura et al. found a significantly lower frequency of anastomotic stricture in the stapled anastomosis group than in the hand-sewn anastomosis group [26].

Hsu et al. compared the operating times entailed in manual and mechanical anastomoses and discovered that duration was significantly shorter in mechanical anastomosis technique than manual method [9]. However, some studies, including meta-analyses, found no significant difference in surgical times between manual and mechanical anastomosis procedures [16, 32]. The results of the present study showed that the effective time of using a stapled technique for cervical esophageal anastomosis was significantly shorter than the manual procedure, This difference may be attributed to a number of reasons: use of numerous hand-sewn techniques described by surgeons (single-layer vs. multilayer anastomosis, interrupted vs. running suture techniques), intraoperative mishaps (e.g., poor alignment of sutures), and the skills of surgeons performing operations [9].

Our research has certain limitations. First, this study was a retrospective study and therefore required clinical trials to achieve acceptable results. Second, the follow-up period spanned 12 months, but a longer time frame may generate different results. Third, all the operations were performed by three surgeons. Although the surgical team had sufficient experience in esophageal surgery and all anastomoses were performed in the neck, the composition of the team may still have resulted in undesirable bias.

## Conclusion

Compared with hand-sewn anastomosis, cervical esophagogastric anastomosis using a side-to-side stapled technique involved a shorter operating time, caused lower anastomotic leakage and stricture rates, and required less frequent anastomotic dilatation. To conclude, we found stapling to be a safer and more superior approach for esophagogastric in patients with esophageal cancer undergoing transhiatal esophagectomy.

## Data Availability

The datasets used and/or analyzed during the current study are available from the corresponding author on reasonable request.
